# Neural network predicts need for red blood cell transfusion for patients with acute gastrointestinal bleeding admitted to the intensive care unit

**DOI:** 10.1038/s41598-021-88226-3

**Published:** 2021-04-23

**Authors:** Dennis Shung, Jessie Huang, Egbert Castro, J. Kenneth Tay, Michael Simonov, Loren Laine, Ramesh Batra, Smita Krishnaswamy

**Affiliations:** 1 Yale School of Medicine, New Haven, CT USA; 2 Computational Biology and Bioinformatics, Yale University, New Haven, CT USA; 3 Department of Computer Science, Yale University, New Haven, CT USA; 4 VA Connecticut Healthcare System, West Haven, CT USA; 5 Stanford University, Palo Alto, CA USA; 6 Department of Genetics, Yale University, New Haven, CT USA

**Keywords:** Gastrointestinal bleeding, Risk factors, Outcomes research

## Abstract

Acute gastrointestinal bleeding is the most common gastrointestinal cause for hospitalization. For high-risk patients requiring intensive care unit stay, predicting transfusion needs during the first 24 h using dynamic risk assessment may improve resuscitation with red blood cell transfusion in admitted patients with severe acute gastrointestinal bleeding. A patient cohort admitted for acute gastrointestinal bleeding (N = 2,524) was identified from the Medical Information Mart for Intensive Care III (MIMIC-III) critical care database and separated into training (N = 2,032) and internal validation (N = 492) sets. The external validation patient cohort was identified from the eICU collaborative database of patients admitted for acute gastrointestinal bleeding presenting to large urban hospitals (N = 1,526). 62 demographic, clinical, and laboratory test features were consolidated into 4-h time intervals over the first 24 h from admission. The outcome measure was the transfusion of red blood cells during each 4-h time interval. A long short-term memory (LSTM) model, a type of Recurrent Neural Network, was compared to a regression-based models on time-updated data. The LSTM model performed better than discrete time regression-based models for both internal validation (AUROC 0.81 vs 0.75 vs 0.75; *P* < 0.001*)* and external validation (AUROC 0.65 vs 0.56 vs 0.56; *P* < 0.001*)*. A LSTM model can be used to predict the need for transfusion of packed red blood cells over the first 24 h from admission to help personalize the care of high-risk patients with acute gastrointestinal bleeding.

## Introduction

Acute gastrointestinal bleeding accounts for over 2.2 million hospital days and 19.2 billion dollars of medical charges annually in the United States and frequently requires red-blood cell transfusion^1^. The management of severe acute gastrointestinal bleeding begins with resuscitation using intravenous fluids and transfusion of packed red blood cells, which are given to 43% of patients hospitalized with upper gastrointestinal bleeding in the United Kingdom and 21% of patients hospitalized with lower gastrointestinal bleeding in the United States^2,3^.

Transfusion needs may change during the hospital stay, but a tool to dynamically predict transfusion needs over time does not yet exist in clinical care. Patients with severe acute gastrointestinal bleeding who require care in the intensive care setting generally have higher transfusion needs and may benefit most from a predictive tool to guide resuscitation efforts. Current guidelines are based on a restrictive transfusion strategy using a hemoglobin threshold of 7 g per deciliter compared to the previous threshold of 9 g per deciliter in patients with upper gastrointestinal bleeding^4^.

**Figure 1:**
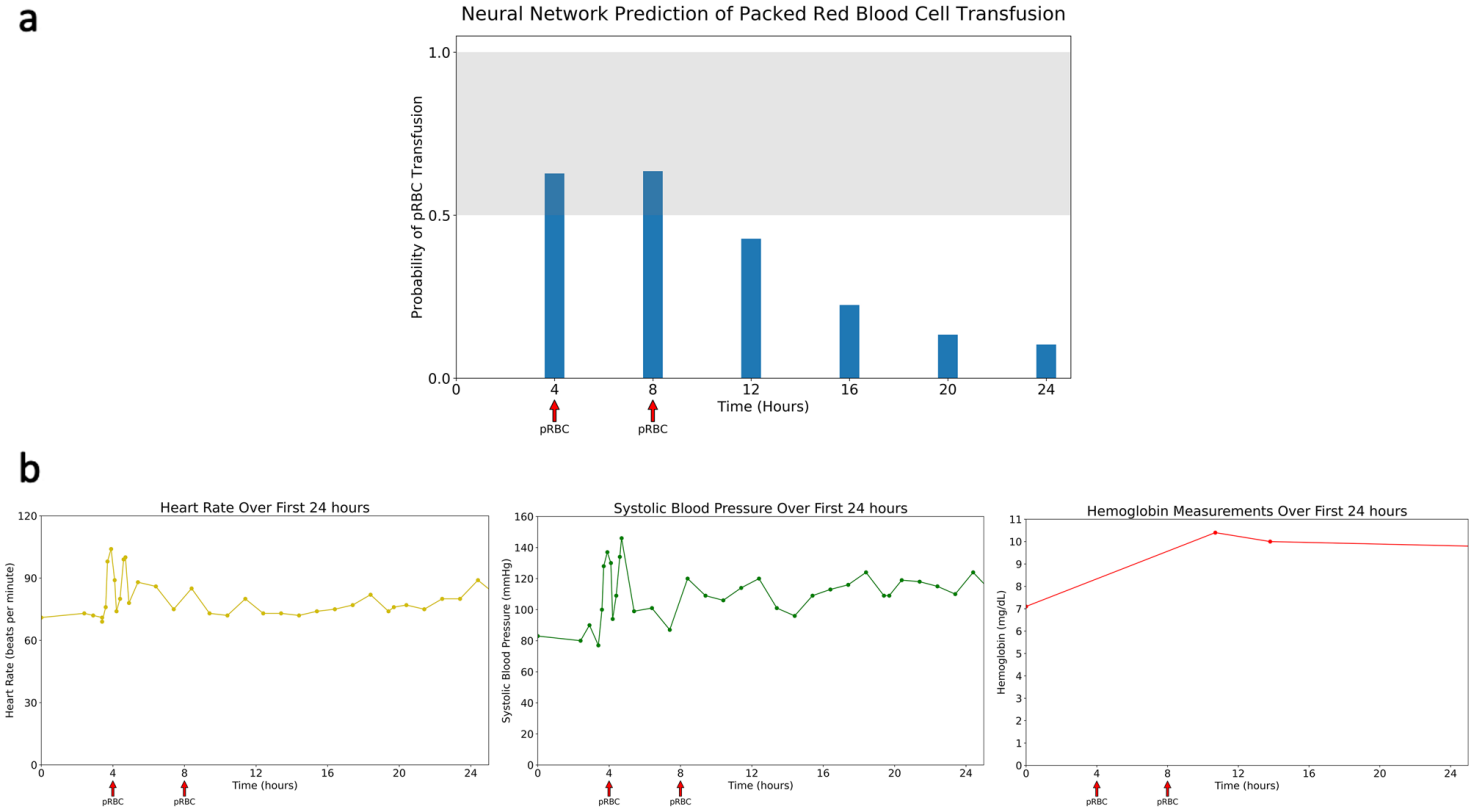
Example of neural network prediction for the first 24 h of a 62 year old man with Hepatitis C cirrhosis presenting with 2 days of intermittent coffee ground emesis and lethargy. Initial Glasgow Blatchford Score = 14 (**a**) Continuous risk prediction of the neural network through the first 24 h with the threshold set above 0.5 for detecting need for transfusion. The arrows indicate need for transfusion during that time period. (**b**) Measurements of Heart Rate, Systolic Blood Pressure, and Hemoglobin occurring during the first 24 h.

Dynamic risk prediction, where predictions are generated in real time every hour based on clinical and laboratory values, may help guide transfusion strategies and help in timing endoscopic intervention, particularly in severely ill patients who require intensive care. Existing clinical risk scores used to screen for risk of needing transfusion of packed red blood cells, such as the Glasgow-Blatchford Score, are static models that only use clinical information at the time of admission (e.g. initial systolic blood pressure)^5^. Machine learning approaches to model risk for gastrointestinal bleeding have shown promise in outperforming existing clinical risk scores, but are also static models^6,7^. Electronic health records (EHRs) can capture clinical data in real time, and have been used to create automated tools to model adverse events, such as sepsis, post-operative complications, and acute kidney injury^8–11^. Recurrent neural networks, a type of neural network that accepts time series data and sequences, have been demonstrated to be better than state-of-the-art risk models for continuous prediction of acute kidney injury up to 48 h, the onset of septic shock 28 h before onset, and all-cause inpatient mortality^12–14^. We propose the use of a Long-Short-Term Memory (LSTM) Network, an advanced recurrent neural network, to process data from electronic health records with an internal memory that stores relevant information over time and can generate a probability of transfusion within the 4 h intervals for patients with severe acute gastrointestinal bleeding. LSTMs have the advantage that feature modules carefully decide what information to store and what information to discard, thereby offering the potential for improved performance. Figure 1 shows the use of our LSTM model in an example patient with generated risk predictions throughout the first 24 h from admission. (Fig. 1).

## Methods

### Data source

A patient cohort presenting with acute gastrointestinal bleeding was identified from the Medical Information Mart for Intensive Care III (MIMIC-III) critical care database^15,16^. The database contains data for over 40,000 patients in the Beth Israel Deaconess Medical Center from 2001 to 2012 requiring an ICU stay. For external validation, a patient cohort presenting with acute gastrointestinal bleeding was extracted from the Phillips eICU Collaborative Research Database (eICU-CRD) of critical care units across the United States from 2014 to 2015. Only urban hospitals with greater than 500 beds were included.

Patients were included if they had an admission diagnosis containing the terms “gastro”, “bleed”, “melena”, “hematochezia”. The diagnoses were collated and then manually reviewed. This inclusion criteria was meant to specifically capture patients with severe acute gastrointestinal bleeding requiring ICU stay. Patients were excluded if vital signs were only available greater than 24 h from time of admission to the ICU, since this constitutes missing values for all 4-h time intervals used to train the models. The data included information that was updated over time during the course of hospitalization, including laboratory results and vital signs. For laboratory values, any negative entry or non-quantizable (e.g. >  = , <) was converted to missing. Medications, current procedural terminology codes, and ICD9/10 codes from the visit were excluded from the analysis. The dataset had a total of 62 features: 5 clinical and demographic variables and 57 laboratory variables.

### Data access

All clinical data from MIMIC-III was approved under the oversight of the Institutional Review Boards of Beth Israel Deaconess Medical Center (Boston, MA). The Phillips eICU Collaborative Research Database (eICU-CRD) was under the oversight of the Massachusetts Institute of Technology (Cambridge, MA). Requirement for individual patient consent was waived by both institutional review boards of Beth Israel Deaconess Medical Center and the Massachusetts Institute of Technology because the project did not impact clinical care and all protected health information was deidentified. All procedures were performed in accordance with relevant guidelines. The data was available on PhysioNet were derived from protected health information that has been de-identified and not subject to HIPAA Privacy Rule restrictions. All use of the data was performed with credentialed access under the oversight of the data use agreement through PhysioNet and the Massachusetts Institute of Technology.

### Study design

The MIMIC cohort included 2524 hospital admissions and was randomly split into a training set with 2,032 hospital admissions and an internal validation set with 492 hospital admissions. (Table 1) We chose to compare the model to a logistic regression model, a standard approach to prediction for time-varying electronic health record data that has previously been applied to acute kidney injury^17^. We also compared the model to a regularized regression model, which uses additional parameters to optimize prediction^18^. The eICU cohort included 1526 hospital admissions from 12 large urban hospitals with over 500 beds. The performance of the neural network model and the regression based models were compared on the internal validation dataset and the external validation dataset.

**Table 1:** Demographics and baseline data for the training and validation set.

	Training set N = 2,032		Validation set N = 492			External validation set N = 1526		
	N	Prop	N	Prop	*p*-value	N	Prop	*p*-value
**Demographic Information**								
Male	836	41%	190	39%	0.31	919	59%	< 0.01
Age								
> 89	144	7%	42	9%	0.29	57	4%	< 0.01
75–89	629	31%	168	34%	0.24	438	28%	0.06
50–75	935	46%	200	41%	0.14	808	52%	< 0.01
25–50	316	16%	71	14%	0.43	211	14%	< 0.01
< 25	8	0%	4	1%	0.27	12	1%	0.13
**Ethnicity**								
White	1429	70%	380	77%	0.08	1246	79%	< 0.01
African American	244	12%	52	11%	0.35	172	11%	0.35
Hispanic	75	4%	22	4%	0.37	27	2%	< 0.01
Asian American	74	4%	15	3%	0.42	20	1%	< 0.01
Other	210	10%	23	5%	0.05	54	3%	< 0.01
**Clinical Features**								
Upper Gastrointestinal Bleeding	679	33%	203	41%	0.07	666	43%	< 0.01
Lower Gastrointestinal Bleeding	428	21%	162	33%	0.02	448	29%	< 0.01
Unspecified Location	925	46%	127	26%	< 0.01	412	27%	< 0.01
**Outcomes**								
Packed Red Blood Cells	1542	76%	381	77%	0.39	515	33%	< 0.01
In-Hospital Mortality	156	8%	32	6.5%	0.35	103	6.6%	0.21

	Mean	Std Dev	Mean	Std Dev	*p*-value	Mean	Std Dev	*p*-value
**Vital Signs**								
Heart Rate (beats per minute)	88.9	18	88.1	16.6	0.35	86.8	17.8	< 0.01
Systolic Blood Pressure	126.9	22.9	127.1	22.2	0.86	119	23.3	< 0.01
Diastolic Blood Pressure	64.2	16.9	65.7	16.6	0.07	61.7	15.4	< 0.01
**Laboratory Tests**								
Alanine Aminotransferase (ALT)	41	86.9	39.8	152	0.87	95.3	272.2	< 0.01
Albumin	3.1	0.63	3.1	0.65	1.00	2.6	0.62	< 0.01
Alkaline Phosphatase	117.2	152.8	118.4	130.5	0.86	136.2	165.2	< 0.01
Anion Gap	15	4.3	14.8	4	0.33	10.6	5.1	< 0.01
Aspartate Aminotransferase (AST)	75.8	159	75	446	0.97	146.6	614.3	< 0.01
Bicarbonate	23.8	4.5	24	4.4	0.37	23.8	5.0	1.0
Bilirubin, Total	1.52	3	1.47	2.5	0.70	4.5	8.0	< 0.01
Calcium, Total	8.1	0.88	8.2	0.8	0.01	8.1	0.76	1.0
Chloride	104.4	6.1	104.4	6.5	1.00	105.6	6.9	< 0.01
Creatinine	1.43	1.2	1.5	1.4	0.31	1.7	1.7	< 0.01
Glucose	147	77.5	143.8	60.6	0.32	128.8	54.1	< 0.01
Magnesium	1.9	0.71	1.9	0.4	1.00	1.9	0.36	1.0
Phosphate	3.5	1.2	3.5	1.2	1.00	3.4	1.4	1.0
Potassium	4.3	0.76	4.4	0.8	0.01	4.0	0.65	< 0.01
Sodium	138.8	4.6	138.7	4.8	0.68	139.2	5.5	0.02
Urea Nitrogen	37.5	28	39.5	30.4	0.18	32.0	26.6	< 0.01
Basophils	0.39	0.98	0.34	0.34	0.06	0.39	0.50	1.0
Eosinophils	1.4	1.9	1.4	1.7	1.00	2.1	2.4	< 0.01
Hematocrit	28	9.5	27.7	6.5	0.41	26.5	4.7	< 0.01
Hemoglobin	9.5	2.5	9.3	2.4	0.10	8.7	1.6	< 0.01
International Normalized Ratio (INR)	1.8	2.5	1.8	1.7	1.00	1.69	1.1	0.14
Lymphocytes (%)	17.1	10.2	16.6	10.1	0.33	15.2	10.1	< 0.01
Mean Corpuscular Hemoglobin (MCH)	30.2	3	29.9	2.9	0.04	29.8	2.4	< 0.01
Mean Corpuscular Hemoglobin Concentration (MCHC)	33.5	1.7	33.3	1.8	0.03	32.3	1.44	< 0.01
Mean Corpuscular Volume (MCV)	90	7.6	89.7	7.3	0.42	90.5	6.3	0.04
Monocytes (%)	4.6	2.6	4.6	2.3	1.00	8.5	5.0	< 0.01
Neutrophils (%)	75.1	12.3	76.5	11.7	0.02	73.3	12.6	< 0.01
Platelet Count (× 1000)	231.8	139.3	238.9	127.4	0.28	174.5	103.8	< 0.01
Prothrombin Time (PT)	17.1	11.4	19.1	15.7	0.01	18.8	10.8	< 0.01
Partial Thromboplastin Time (PTT)	31.3	13.7	31.3	14.2	1.00	39.9	20	< 0.01
Red Blood Cell Distribution Width (RDW)	16.1	2.5	15.9	2.2	0.08	17.1	2.8	< 0.01

### Input variables

A total of 62 input variables were used and included age, gender, vital signs (systolic blood pressure, diastolic blood pressure, heart rate), and 57 unique laboratory values. (Table 2) The vital signs and laboratory values were extracted and then consolidated into 4-h time intervals over the first 24 h from admission. These features were selected because they reflect dynamic changes from measurement in the ICU; ICD codes and CPT codes associated with the encounters were not included since they are not available at the time of care provision and therefore not available in real time for prediction. Medications have different formulations, with no clear definition of relevant medication types or standardization across multiple centers and were not included as features for this analysis.

**Table 2:** Input variables (N = 62).

Category	Input Variables
Demographic (2)	Gender Age
Vital Signs (3)	Heart Rate Systolic Blood Pressure Diastolic Blood Pressure
Laboratory variables (57)	Blood Gas (Base Excess, Total Carbon Dioxide, Oxygen Saturation, pH, Arterial Pressure of Oxygen) White Blood Cells, Neutrophils, Basophils, Eosinophils, Lymphocytes, Bands, Monocytes, Hemoglobin, Hematocrit, Mean Corpuscular Hemoglobin, Mean Corpuscular Hemoglobin Concentration, Mean Corpuscular Volume, Red Blood Cell Distribution Width, Platelet Count, International Normalized Ratio, Prothrombin Time, Partial Thromboplastin Time Sodium, Potassium, Chloride, Bicarbonate, Anion Gap, Magnesium, Phosphate, Calcium, Creatinine, Urea Nitrogen, Glucose Alanine Aminotransferase, Aspartate Aminotransferase, Alkaline Phosphatase, Albumin, Amylase, Lipase, Direct Bilirubin, Total Bilirubin Creatine Kinase, Creatine Kinase-MB, Ferritin, Total Iron, Iron Binding Capacity, Lactate, Lactate Dehydrogenase, Thyroid Stimulating Hormone, Transferrin, Troponin T, Vancomycin, Fibrinogen Urine Studies (Creatinine, Sodium, Specific Gravity)

### Outcome variable

The predicted outcome measure was the transfusion of packed red blood cells, calculated as binary 0 (no transfusion) or 1 (transfusion given). At the beginning of each 4-h time interval, the model makes a prediction on whether a transfusion will be needed at the next 4-h interval.

### Data pre-processing

Each patient encounter was represented by a sequence of events with each 4-h period containing information recorded in the vitals and laboratory values. Information for each patient encounter was encoded into 4-h time intervals up to the first 24 h. After excluding lab values with greater than 90% missingness, remaining lab values with greater than 50% missingness in the dataset were converted to missing indicator variables, with 1 as present and 0 as missing. To harmonize the input variables across patients, the first timepoint for each patient encounter was fixed at the first recording of heart rate, systolic blood pressure, and diastolic blood pressure. Consolidation of vital signs and laboratory values in each 4-h interval was performed by taking the mean of each value. All continuous values were normalized and centered. Age was maintained as a continuous variable, with patients greater than 89 years old coded as 89 years old. After consolidation, 86% (1651/1923) of the encounters had information for every 4-h interval in the full 24 h period. For the training set 7% of the 4-h periods (855/13,167) were labeled as receiving a packed red blood cell transfusion, the test set 4% (134/3149), and the external validation set 2% (157/8414). In summary, each patient encounter has up to 6 predictions for a total of 6*n predictions in the entire dataset, and we compute one ROC curve and associated AUC for this total. This ensures that the same threshold exists across every time period.

### Missing values

To examine the role of the data imputation method used, we compared 4 different imputation strategies. The first was imputation of the mean value for any missing value. The second was a carryforward approach, or using the previously recorded value if a value was present at a previous time point but no subsequent value was measured. This assumes that the laboratory value is constant until the next time point in clinical decision-making^19^. The third was mean imputation with a new variable that served as a missingness indicator for every variable. The fourth was carryforward with a missingness indicator for every variable.

### LSTM neural network model background

**Figure 2:**
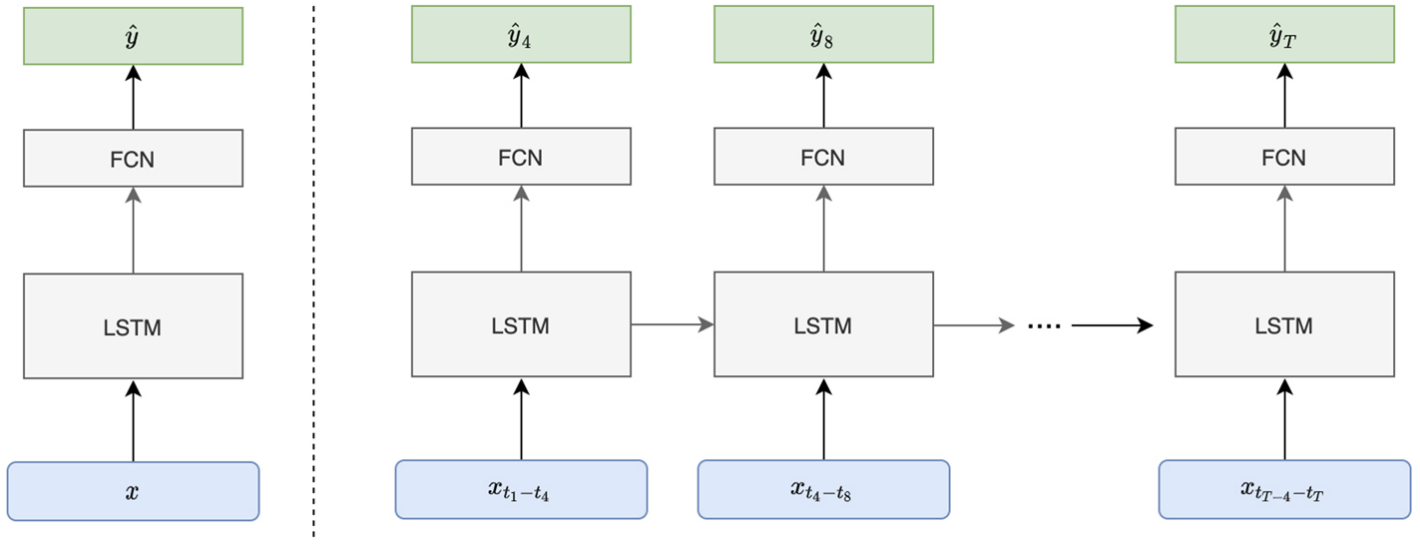
Long-Short Term Memory (LSTM) Network Model Overview. Electronic Health Record data (vitals, laboratory values) is fed into the model, passed through the layers, transformed, and gives a probability of the outcome (transfusion of packed red blood cells). At the beginning of each 4-h interval the LSTM Network can generate a probability of needing transfusion. T represents the time in hours, X represents input data (vitals, laboratory values), Y represents the probability of needing transfusion, and FCN is a fully convolutional network that processes the information from the previous time period to generate the prediction.

Recurrent neural networks allow for processing of sequential information by storing information as internal states over multiple time points. Long short-term memory (LSTM) networks are a type of RNN that can be useful for clinical measurements because they carefully tune the information passed between subsequent time-iterations of the model (Fig. 2). The LSTM has a single output that serves as a prediction and other hidden states that are then fed back into the neural network to adjust the final output. For the implementation of the model, we used the PyTorch deep learning library. Given a series of EHR data, ${{\varvec{x}}}^{\left(0\right)}, {{\varvec{x}}}^{\left(1\right)},\dots , {{\varvec{x}}}^{\left({\varvec{T}}-1\right)}$, where ${{\varvec{x}}}^{\left({\varvec{t}}\right)}$ represents the input variables for the $(t+1)$ th 4-h interval, at the beginning of each 4-h interval our goal is to predict whether transfusion is needed in the next 4 h. The output is a sequence of probability predictions $\widehat{{y}^{\left(1\right)}},\widehat{{y}^{\left(2\right)}},\dots ,\widehat{{y}^{T}}$, where $\widehat{{y}^{\left(t\right)}}\in \left[\mathrm{0,1}\right]$ is the prediction for whether transfusion is needed in the *t*th 4-h interval. The LSTM model consists of 2 layers of 128 LSTM cells each, followed with a linear layer that maps from hidden state space to the prediction space. We obtain the log-probabilities by adding a LogSoftmax later in the last layer of the network. Thus the output of the neural network is a sequence $\widehat{{{\varvec{p}}}^{\left(1\right)}},\widehat{{{\varvec{p}}}^{\left(2\right)}},\dots ,\widehat{{{\varvec{p}}}^{\left(T\right)}}$, where $\widehat{{{\varvec{p}}}^{\left(t\right)}}$ is the log-probability of $\widehat{y}$ being either of the target classes, and our decision rule is to administer transfusion if $\widehat{{{\varvec{p}}}^{\left(t\right)}}>threshold$, where the threshold is determined by desired sensitivity or specificity. We use the negative log likelihood for the output at each time of interest as the loss function. The model is trained for up to 100 epochs with hyperparameters corresponding to the lowest validation loss recorded and used to obtain testing accuracy.

### Discrete time logistic regression and regularized regression

For comparison discrete-time regression approaches were employed to generate a new prediction using each 4-h block of data to predict the need for transfusion for the next 4-h block of data. We used both logistic regression and regularized regression with elastic net penalty using the glmnet package in R tuned by fivefold cross-validation on the training set (Appendix A). The training protocol was to take every 4-h sequence and then using all the 4-h sequences to train the regression models, since the model is designed to generate a prediction for any 4-h sequence. The same covariates were used that were available for the LSTM neural network model at each 4-h time interval, with no additional features used to train the model. The different imputation strategies as described previously were also employed.

#### Statistical analysis

Two-tailed t tests and chi-squared test were used to compare baseline characteristics between the training and validation sets. We assessed model performance using the area under the curve (AUROC) and compared it to the performance of logistic regression using the nonparametric DeLong test^20^. Confidence intervals were calculated with 2000 stratified bootstrap replicates. McNemar’s test was used to compare the optimal sensitivity and specificity threshold by the Youden Index.

## Results

Demographics were similar between training and internal validation sets with the median age 69 for both, proportion of men (41% in training, 39% on internal validation), and predominantly white (70% in training, 77% in internal validation). There was a similar percentage of patients with upper gastrointestinal bleeding (training 33% vs internal validation 41%), but the training set had more patients with gastrointestinal bleeding from an unspecified source (46% vs 26% *P* < 0.01), while the internal validation set had more patients with lower gastrointestinal bleeding (33% vs 21% *P* = 0.02). Vital signs and laboratory values were similar in the training and internal validation sets. (Table 1) The external validation set was significantly different from the training and internal validation with demographics notable for a generally younger population, increased patients with upper and lower gastrointestinal bleeding and less patients with an unidentified source. Furthermore, the transfusion rate was significantly lower (33% versus 76%; *P* < 0.01), reflecting modern guidelines of restrictive transfusion strategy for the treatment of acute gastrointestinal bleeding. Laboratory tests were notable for decreased hemoglobin and hematocrit, increased ALT, AST, alkaline phosphatase and total bilirubin, increased creatinine and decreased albumin. (Table 1).

**Figure 3:**
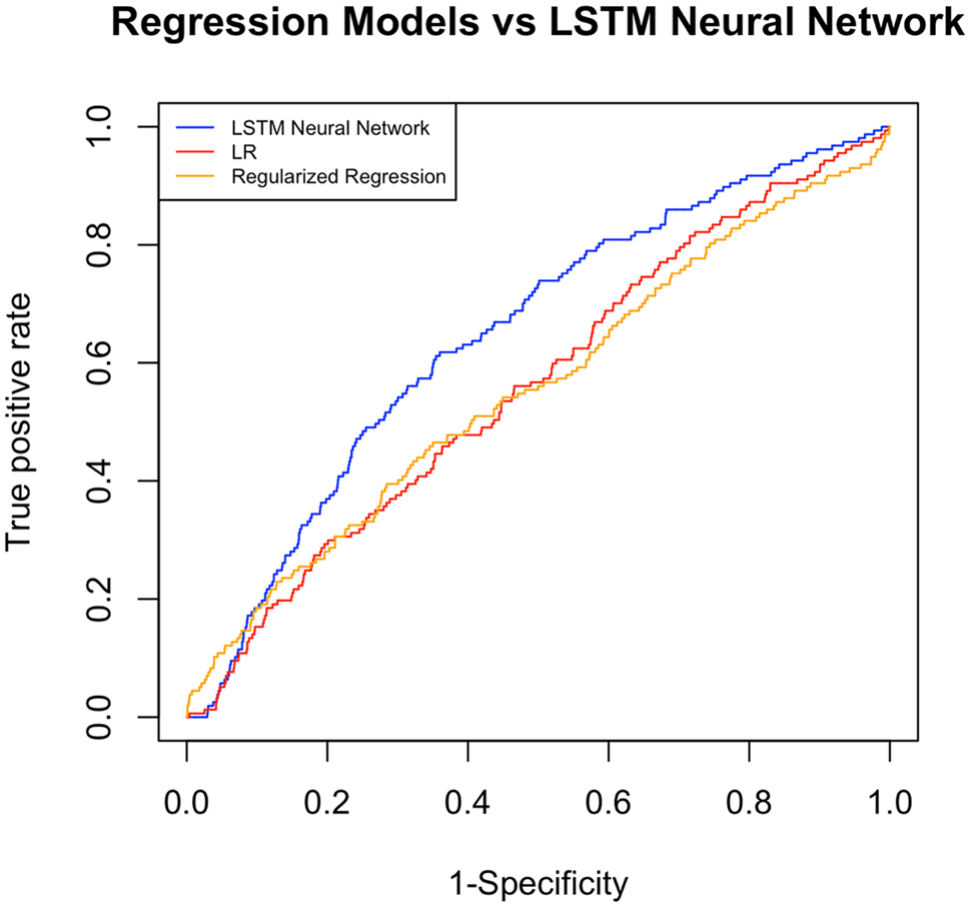
Comparison on external validation of the overall Area Under the Receiver Operating Curve (AUROC) as a measure of performance of the Long-Short Term Memory (LSTM) Neural Network model, discrete time Logistic Regression (LR), and regression with elastic net penalty.

The performance of the LSTM model on the four different imputation strategies were similar and all significantly better than the discrete time logistic regression model. (Table 3) The results we subsequently present are for the strategy with the highest AUROC (carryforward and missing indicators). For the main analysis of all patients with acute gastrointestinal bleeding who were transferred to the ICU, the LSTM performed significantly better than both regression-based approaches. On internal validation, the LSTM outperformed LR (AUROC 0.81 CI 0.80–0.83 vs 0.75 CI 0.73–0.77; *P* < 0.001) and regularized regression (AUROC 0.81 CI 0.80–0.83 vs 0.75 CI 0.73–0.78; *P* < 0.001) in predicting packed red blood cell transfusion across the entire 24 h period. For external validation, the LSTM outperformed LR (AUROC 0.65 CI 0.61–0.69 vs 0.56 0.51–0.60; *P* < 0.001) and regularized regression (AUROC 0.65 CI 0.61–0.69 vs 0.56 0.52–0.61; *P* < 0.001). (Table 4, Fig. 3).

**Table 3:** Performance of the Long-Short Term Memory (LSTM) Model and the discrete time Logistic Regression (LR) model in Predicting Transfusion of Packed Red Blood Cells by Comparison of Area Under the Receiver Operating Curve (AUROC) for Internal Validation (N = 492) and External Validation (N = 1526).

	Long-short term memory network model AUROC 95% CI	Logistic Regression AUROC 95% CI	*p*-value	Regularized logistic regression with elastic net AUROC 95% CI	*p*-value
Internal Validation	0.81 (0.80–0.83)	0.75 (0.73–0.77)	< 0.001	0.75 (0.73–0.78)	< 0.001
External Validation	0.65 (0.61–0.69)	0.56 (0.51–0.60)	< 0.001	0.56 (0.52–0.61)	< 0.001

**Table 4:** Comparison on external validation only of the overall performance of Long-Short Term Memory network model compared to the Logistic Regression model with different imputation methods to address missingness in the first 24 h after admission for all patients admitted to the Intensive Care Unit with Acute Gastrointestinal Bleeding.

External validation set	LSTM AUROC 95% CI	Logistic regression AUROC 95% CI	*p*-value	Regularized logistic regression with elastic net penalty AUROC 95% CI	*p*-value
Mean Imputation	0.65 (0.60–0.69)	0.54 (0.49–0.59)	< 0.001	0.55 (0.50–0.60)	< 0.001
Carryforward Imputation	0.66 (0.62–0.70)	0.56 (0.51–0.60)	< 0.001	0.56 (0.51–0.60)	< 0.001
Mean Imputation and Missing Indicators	0.64 (0.60–0.68)	0.54 (0.49–0.59)	< 0.001	0.55 (0.50–0.60)	< 0.001
Carryforward Imputation and Missing Indicators	0.65 (0.60–0.69)	0.56 (0.51–0.60)	< 0.001	0.56 (0.52–0.61)	< 0.001

### Sensitivity and specificity cutoff

The optimal sensitivity and specificity cutoff was obtained using Youden’s index and was found on external validation for the LSTM neural network to be 62% sensitivity and 64% specificity; the logistic regression optimal cutoff was 47% sensitivity and 65% specificity (*P* < 0.001).

### Sensitivity analysis

Sensitivity analysis was performed on the external validation dataset by gender, age, systolic blood pressure, blood urea nitrogen, and hemoglobin, variables commonly used in assessing risk for patients with acute gastrointestinal bleeding. When subset by gender the LSTM model still outperformed the LR model (0.64 vs 0.54, *P* = 0.002) and the regularized regression model (0.64 vs 0.49; *P* = 0.02). In the subset of patients with age greater than 65, which was the mean of patients with acute gastrointestinal bleeding, the LSTM model outperformed the LR model (0.61 vs 0.54, *P* = 0.008) and the regularized regression model (0.61 vs 0.56; *P* = 0.01). For vital signs and laboratory values, cutoffs were derived from the Glasgow Blatchford Score: systolic blood pressure cutoff less than 110 mmHg, blood urea nitrogen greater than 18.2, and hemoglobin less than 10 (similar risk category for both men and women). The LSTM model outperformed the LR and regularized regression models in all these analyses. (Table 5) Sensitivity analyses of the opposite group (men only, patients less than 65 years old, and the lower risk cutoff for vital signs and laboratory values) are provided in Appendix B.

**Table 5:** Sensitivity Analyses for external validation eICU dataset. Systolic Blood Pressure, BUN, and hemoglobin cutoffs were derived from the Glasgow Blatchford Score.

Total encounters N = 1526	LSTM neural network	Logistic regression	*p*-value	Regularized logistic regression with elastic net	*p*-value
Female N = 607	0.64 (0.57–0.71)	0.54 (0.46–0.62)	0.002	0.49 (0.42–0.56)	0.02
Age > 65 (mean) N = 820	0.61 (0.55–0.67)	0.54 (0.47–0.60)	0.008	0.56 (0.50–0.61)	0.01
Systolic Blood Pressure < 110 N = 849	0.64 (0.58–0.69)	0.57 (0.50–0.63)	0.03	0.55 (0.49–0.61)	0.002
BUN > 18.2 N = 1110	0.64 (0.58–0.69)	0.56 (0.50–0.62)	0.003	0.56 (0.50–0.61)	< 0.001
Hemoglobin < 10 N = 1303	0.64 (0.60–0.69)	0.56 (0.51–0.62)	0.001	0.56 (0.51–0.61)	< 0.001

Hemoglobin cutoff was chosen due to the matched risk for both men and women.

## Discussion

Predicting the need for transfusion of packed red blood cells has direct relevance to guiding the management of patients with acute gastrointestinal bleeding. This is the first study to show that a LSTM network model is able to predict the need for packed red blood cell transfusion for patients with severe acute gastrointestinal bleeding with superior performance to time-varying logistical regression with internal and external validation. By anticipating needs for transfusion, this is a first step towards personalizing treatment and tailoring appropriate resuscitation to reduce clinical decompensation and death for patients with severe acute gastrointestinal bleeding. While endoscopic evaluation is important, adequate resuscitation is an important part of management prior to endoscopy^21–24^.

In this work we use a (one-directional) 2-layer LSTM with 128 hidden units in each layer. The LSTM setup is a commonly used variation of the LSTM which consists of the original LSTM architecture with added forget gates and full gradient backpropagation through time (BPTT) training^25,26^. We use this model over a simple recurrent neural network (SRNN) as it addresses weaknesses inherent in SRNNs such as difficulty learning dependencies across multiple time steps and aberrant gradient flow. A comparative study of LSTM variants concluded that while many variations of LSTMs exist, much of the improved performance can be attributed to forget gates and the choice of activation function^27^. Advantages of the LSTM over regression models include the ability to generate multiple predictions with the first data input and the ability to combine features in more complex ways to model changes over time. The trained architecture can be used to generate predictions for each time period using presenting data from the first 4 h, whereas the regression models have fixed coefficients that can only generate predictions as data becomes available for each time period. For example, for a patient admitted to the ICU with data from the first 4 h, the LSTM neural network can propagate the data through its architecture to predict need for transfusion at 8, 12, 16, 20, and 24 h. Using regression models, it could only be used to predict the need for transfusion at the next time period. While regression models use weighted sums of features with specific thresholds for prediction, neural networks can combine features in non-linear and more complex ways to generate predictions.

Previous risk scores capture information from specific points in time at admission, and do not incorporate new clinical data over the course of hospitalization. Electronic health records contain longitudinal information on patients admitted to the hospital and reflect real-world practice, which can be used to develop risk prediction models^28^. For patients who have severe disease requiring intensive care unit stay, mortality may be due to end organ damage from inadequate perfusion; this dynamic risk prediction can potentially optimize transfusion timing to improve overall organ perfusion^3, 29,30^. Despite the significant computing requirements necessary to run neural networks, existing electronic health records are now deploying cloud computing infrastructure able to perform computationally intensive tasks. The emerging capabilities of cloud infrastructure in electronic health records, such as the Cognitive Computing platform for Epic Systems, make the deployment of neural networks for clinical care feasible.

We envision the future of care for all patients to be enhanced by customized machine learning decision support tools that will provide both initial risk stratification and ongoing risk assessment to provide treatment at the right time for the right patient. Using a dynamic risk assessment, resuscitation needs could be estimated early and optimized in preparation for endoscopic evaluation and intervention. This individualized decision-making potentially will minimize organ damage from inadequate resuscitation, which drives the risk for mortality in these patients^29^. The LSTM model can be tuned for provider preference. Alert fatigue is particularly relevant in the ICU, since clinically irrelevant alerts can have an impact on patient safety^31^. In order to minimize alert fatigue, a high specificity threshold could be set for the algorithm. However, if providers do not want to miss any time periods when patients need packed red blood cell transfusions, a high sensitivity threshold can be set to minimize false negatives. Although the LSTM network model is much better than a standard regression-based approach, it still falls short of optimal performance. More work will be needed to develop and validate neural network models.

Interpretability is a key area of active research for neural network models, particularly in order to assess the trustworthiness of the prediction. Approaches attempt to elucidate the hidden states of the network architecture, identify features important to prediction, and perform saliency analyses to identify input data most relevant to the model prediction^32–35^. Another approach attempts to learn an interpretable model around the prediction, called Local Interpretable Model-agnostic Explanations (LIME)^36^. These approaches, however, should be filtered through the usefulness for a front-line clinician who has both prior knowledge about the application and the ability to reason through the available evidence after receiving the prediction. As professionals with authority due to training and experience, clinicians may benefit less from the “hidden states” and more from presenting the relative importance of input variables; the latter allows for clinicians to assess the prediction as plausible or due to confounding^37^. Applying these techniques is outside the scope of this manuscript and will be explored in future work.

Strengths of this study include external validation in a more recent ICU electronic health record dataset and modeling patients with severe illness requiring intensive care unit stay, which may benefit disproportionately from timely transfusion and resuscitation and the use of vital signs and laboratory tests that are standardized and can be easily mapped across electronic health record systems. Our comparison to regression models is stronger than a comparison to currently used clinical scores such as the Glasgow-Blatchford Score or Oakland Score, which were developed to generate a static risk prediction with only data at presentation.

Limitations include the absence of prospective and independent validation in other electronic health record-base datasets. Despite showing external validation on a temporally and geographically separate dataset of patients with acute gastrointestinal bleeding requiring ICU care, prospective validation and implementation into clinical practice is crucial to quantifying the benefit of such systems on patient outcomes. Additionally, the performance difference between test set and validation set may be due to the lower prevalence of packed red blood cell transfusions in the external validation set, which may indicate need for re-training of the model with more updated clinical data that reflect the decreased use of transfusions. The definition of ground truth is the receipt of a transfusion, and not on the judgment of whether they should have received a transfusion, which may not reflect the current standard of care and may not be applicable to hospitals that are resource limited. The use of encounters as independent episodes rather than individual patients may lead to bias and information leak, particularly since there are around 708 patients with more than one encounter for severe acute gastrointestinal bleeding requiring ICU care. However, the decision was made to include all encounters for these patients to reflect real world practice since the bias is tolerable from a clinical standpoint: patients with recurrent severe acute gastrointestinal bleeding requiring ICU care are the very patients who would stand to benefit from these predictions. We also control for information leak since all features except for age and sex and unique for each ICU encounter. Comparison with regression-based models may change if the models incorporate aggregated data available at time of predictions from previous time intervals (e.g. the mean and standard deviation) and should be explored in future studies. In addition, the segmentation into 4 h segments may lead to distortions, since the same signal of transfusion can be administered immediately after bound of the 4-h time interval or several hours afterwards (e.g. 5 min or 2 h afterwards). Additionally, the proportion of missing data required imputation, which may introduce bias to the data. To quantify the difference, we compared different imputation strategies including carryforward and found no clear difference in the overall performance of the models.

In summary, we present the first application of recurrent neural networks to dynamically predict need for packed red blood cell transfusion over time using electronic health record data. We report superior performance compared to a discrete time regression models. Our approach may lead to delivery of earlier resuscitation with packed red blood cells to minimize ischemic end organ damage in patients with severe acute gastrointestinal bleeding. Future directions include external validation of the model on other cohorts of high-risk patients with gastrointestinal bleeding, along with prospective implementation and deployment in the electronic health record system for high-risk patients with gastrointestinal bleeding.

## Data availability statement

Code used to generate the dataset will be made available for review at https://github.com/dshung.

## Appendix B

Sensitivity Analyses for external validation eICU dataset. The counterfactual subgroups of the Systolic Blood Pressure, BUN, and hemoglobin cutoffs derived from the Glasgow Blatchford Score are presented here. The hemoglobin cutoff was chosen due to the matched risk for both men and women.

**Table:**

Total encounters N = 1526	LSTM neural network	Logistic regression	*p*-value	Regularized logistic regression with elastic net	*p*-value
Male N = 919	0.64 (0.57–0.71)	0.60 (0.54–0.66)	0.09	0.60 (0.55–0.66)	0.05
Age < 65 (mean) N = 706	0.68 (0.62–0.75)	0.62 (0.55–0.70)	0.05	0.57 (0.50–0.64)	< 0.001
Systolic Blood Pressure > 110 N = 846	0.70 (0.64–0.76)	0.64 (0.57–0.70)	0.02	0.62 (0.56–0.69)	0.001
BUN < = 18.2 N = 442	0.66 (0.56–0.76)	0.59 (0.47–0.71)	0.06	0.55 (0.44–0.66)	0.01
Hemoglobin > = 10 N = 542	0.67 (0.59–0.76)	0.56 (0.46–0.66)	0.01	0.55 (0.46–0.64)	0.006

## Abbreviations

RNN: Recurrent Neural Network
LSTM: Long-Short Term Memory
LR: Logistic Regression
HER: Electronic Health Record
MIMIC-III: Medical Information Mart for Intensive Care III
AUROC: Area Under the Receiver Operating Curve
